# The Steiner tree Prosecutor: Revealing and disrupting criminal networks through a single suspect

**DOI:** 10.1371/journal.pone.0312827

**Published:** 2024-12-02

**Authors:** Fredy Troncoso, Richard Weber

**Affiliations:** 1 Departamento de Ingeniería Industrial, Facultad de Ingeniería, Universidad del Bío-Bío, Concepción, Chile; 2 Departamento de Ingeniería Industrial, FCFM, Universidad de Chile, Santiago, Chile; 3 Instituto de Sistemas Complejos de Ingeniería (ISCI), Santiago, Chile; National Institute of Genomic Medicine: Instituto Nacional de Medicina Genomica, MEXICO

## Abstract

Disrupting a criminal organization requires a significant deployment of human resources, time, information, and financial investment. In the early stages of an investigation, details about a specific crime are typically scarce, often with no known suspect. The literature has shown that an effective approach for analyzing criminal organizations is social network analysis. This approach allows the use of traditional social network tools for analyzing criminal networks, as well as more sophisticated and recent tools. This article introduces a model called *StPro*, which enables the identification of members of a criminal organization starting from a single suspect. It utilizes linear optimization modeling based on Steiner trees. A suspect is used as the root node, and the resulting tree reveals a probable configuration of the criminal organization to which the suspect may belong. Its application to a real-world case demonstrates that there are no significant differences in effectiveness between the proposed model and the state-of-the-art in the literature, despite requiring less information. It also demonstrates how its application aided in the identification of a gang dedicated to violent crimes in Chile. These results highlight the strong capability of the proposed model to support criminal investigations.

## 1 Introduction

A criminal group is defined as a structured group formed by two or more people and that is characterized by serious criminal activity over time, with high internal cohesion, and a hierarchical and specialized structure [1].

The structure of a criminal group is given by the relationships between its members and is fundamental for the success of its operations. It is also key in police investigation since it allows the identification of a criminal gang among a group of suspects as well as of key individuals within such a gang [2].

Criminal groups are aware of the importance of concealing both their members and the relationships between them. However, they face an inherent dilemma between keeping this structure secret and maintaining the operational capacity necessary to effectively coordinate and control their members [3].

Disrupting these groups is essential to weakening their operations and capturing key individuals. To achieve this, it is crucial to have access to critical information that allows for the identification of those members whose removal would cause the greatest disruption to the organization [4].

The transformation of data into graphs is a crucial step in criminal investigations, as it allows criminal networks to be represented mathematically through nodes (individuals) and edges (relationships). However, this data conversion, often ambiguous and complex, can be labor-intensive and relies on the analyst’s judgment, presenting significant challenges such as ‘fuzzy boundaries’ within criminal networks [5]. Despite these obstacles, police investigations generate key data to estimate relationships between criminals from four main sources: self-reports from individual actors, evidence of communication or resource transfers, similarities in social behavior, and observations of joint participation in events [6]. These relationship estimates enable the application of graph-based approaches, where individuals are represented as nodes and relationships as edges [7, 8]. Such approaches have been instrumental in the investigation of criminal groups for over three decades [9], providing a more structured understanding of the connections within criminal networks.

The most widely used tools in this context include social network analysis (SNA) centrality measures, such as degree, closeness, betweenness, and eigenvectors [10], which help to quantify the influence of individuals within the network. Specific techniques have also been developed to analyze, identify, and visualize criminal groups, aiding in the identification of key individuals whose roles are pivotal to the network’s functioning [11–13].”

Within the search techniques for criminal groups, association searches between two suspected individuals serve as an effective tool [14–16]. These association searches aim to uncover routes between two suspects who initially appear unrelated, potentially revealing hidden connections. Individuals within these routes are considered potential members of the criminal group and are prioritized in police investigations, providing additional insight into key individuals whose removal could further disrupt the organization.

However, in most cases, criminal groups commit crimes without identifiable suspects, so having even one suspect is a critical starting point for any investigation. To address this challenge, the objective of this research is to identify members of a criminal group starting from a known individual. A model is proposed that facilitates this search through a tree structure rooted in the suspect. This method enables investigators to systematically trace connections, uncover hidden members within the group, and provide a robust tool for mapping out the criminal network. Our work introduces an integer linear programming model based on the single-point weighted Steiner tree problem or the rooted prize-collecting Steiner tree problem [17].

The remainder of this article is organized into four sections. Section 2 provides the theoretical foundations on which the proposed model is based. Section 3 introduces the proposed model, which uses integer linear programming to identify a criminal gang using only one suspect. Section 4 applies the proposed model to a real-world case of crime investigation using historical data, thus highlighting its versatility. In Section 5, we show how our model identified gang members during an ongoing police investigation. Finally, Section 6 presents our conclusions and some suggestions for further development.

## 2 Background

Section 2.1 presents the node-weighted Steiner tree problem which will be used in Section 3 to identify criminal groups starting with only one suspect. Section 2.2 presents literature relevant to the definition of the utility function to be used in the proposed model.

### 2.1 Node-weighted steiner tree problem

The Steiner Tree problem (*STP*) in graphs is a combinatorial optimization problem that has been widely used in network design, integrated circuit design, localization problems, machine learning, systems biology, and bioinformatics [18]. In general, the *STP* seeks a tree that interconnects a set of nodes *S* called terminals at a minimum cost [19].

To describe the *STP*, we consider *G*(*N*, *E*), an undirected graph consisting of a set of nodes *N* = *S* ∪ *T*, where *T* is a set of potential Steiner nodes and a set of edge *E*. Each edge (*i*, *j*) ∈ *E* has an associated cost *c*_*ij*_ > 0 [20]. The graph *G*′(*N*′, *E*′) represents an *STP* solution if it meets the following conditions:

- *G*′(*N*′, *E*′) is a tree.
- *E*′ ⊆ *E*.
- *N*′ = *S* ∪ *T*′, where *T*′ ⊆ *T*.
- The total cost in the edges of *E*′ is the minimum of all the trees that meet the above conditions.

A natural extension of the *STP* is the so-called node-weighted *STP* which considers the nodes’ weights or costs *c*_*i*_ < 0 for every node *i* ∈ *T* and *S* = {*r*}, for an *r* ∈ *N*. To describe the problem in which the sum of costs associated with the nodes and the costs of the edges is minimized, we consider the following decision variables:

$$\begin{array}{c} X_{ij}=\{\begin{array}{cc} 1 & \text{if}\;\left(i,j\right)\;\;\in E^{\prime }\\ 0 & \text{otherwise}\; \end{array} \end{array}$$ (1)

$$\begin{array}{c} Y_{i}=\{\begin{array}{cc} 1 & \text{if}\;i\;\;\in N^{\prime }\\ 0 & \text{otherwise}\; \end{array} \end{array}$$ (2)

$$\begin{array}{c} f_{ij}=\text{flow}\;\text{on}\;\text{edge}\;\text{(i,j)} \end{array}$$ (3)

The formulation of the node-weighted STP is as follows:

$$\begin{array}{c} Min\;\;\sum_{i\in T}c_{i}Y_{i}+\sum_{(i,j)\in E}c_{ij}X_{ij} \end{array}$$ (4)

s.t.

$$\begin{array}{c} \sum_{i\in N}X_{ij}=Y_{j}\;\forall j\in T \end{array}$$ (5)

$$\begin{array}{c} \sum_{i\in N}\left(f_{ij}-f_{ji}\right)=Y_{j}\;\forall j\in T \end{array}$$ (6)

$$\begin{array}{c} f_{ij}\leq \left(|N|-1\right)X_{ij}\;\forall i\in N,j\in T \end{array}$$ (7)

$$\begin{array}{c} Y_{r}=1 \end{array}$$ (8)

$$\begin{array}{c} X_{ij},Y_{i}\in \{0,1\},f_{ij}\geq 0\;\forall i\in N,j\in T \end{array}$$ (9)

The objective function in Eq (4) accounts for the total cost of all nodes and edge that belong to the tree. Constraint (5) ensures that if node *j* is in the tree, one edge enters it. Eq (6) assures the conservation of the flow and constraint (7) prevents cycles. Constraints (5) and (6) ensure a tree structure. Constraint (8) establish the root node and constraint (9) consists of binary and non-negativity constraints.

In some formulations, it is common for the weighting of the nodes to represent the benefits *r*_*i*_ and for the edges to represent the costs *c*_*ij*_. In this case, the objective is to determine a sub-tree minimizing the cost or maximizing the revenue (or profit), subject to restrictions. In some problems, both the cost and the revenue are combined in the objective function, and in others, the cost or income is limited by restrictions. Depending on the problem, some of these criteria can be ignored or combined [21]. When the real profit of the objective function, as in Eq (10), is used the problem is known as the net-worth maximization problem [21, 22].

$$\begin{array}{c} Profit=\;\;\sum_{i\in T}r_{i}Y_{i}-\sum_{(i,j)\in E}c_{ij}X_{ij} \end{array}$$ (10)

When *S* contains only one element, we get the special case called the single point weighted Steiner tree problem *SPWSTP* or rooted prize-collecting Steiner tree problem *RPCSTP* [22]. In Section 3, we use this kind of model.

### 2.2 Utility function of a crime planner

The search for a criminal group can be seen as an associations process by which a crime planner *s* plans a group crime by choosing other criminals. The planner is rational and chooses criminals with the skills and trustworthiness that guarantee that the crime is carried out and that it is done with the maximum utility. Criminal skills are represented by the criminal propensity *Pcg* and trustworthiness through the social distance between individuals *d*_*ij*_ [16]. To determine the criminal propensity of individual *i* (*Pcg*_*i*_), we consider the available information regarding individual *i* at the time of launching the investigation. The general form for estimating *Pcg*_*i*_ is given by:

$$\begin{array}{c} Pcg_{i}=f\left(s_{i}\right)\;\;\;\forall i\in N \end{array}$$ (11)

where *s*_*i*_ is the set of relevant attributes of individual *i* and *f* is a function chosen under a certain context that transforms these attributes in a context-dependent manner into a propensity value.

The social distance between two individuals is commonly represented by a value between 0 and 1, where 1 represents the maximum distance between them. The sources of information for estimating a representative value for the distance are different in each application.

Derived from the utility functions of criminal organizations proposed by [23–26], the following utility function is proposed for a crime planner given a network *G*(*N*, *E*) [14]:

$$\begin{array}{c} U=I\;Pr\left(Pcg_{i};i\in A\right)-\sum_{i\in E}W_{i}\left(Pcg_{i}\right)-C\;Pr\left(\sum_{\left(i,j\right)\in E_{A}}d_{ij}\right) \end{array}$$ (12)

where *I* is the income from committing the crime; the term *Pr*(*Pcg*_*i*_;*i* ∈ *A*) represents the probability of committing the crime, which depends on the criminal propensity *Pcg*_*i*_ of the individuals *i* that belong to the association *A* ⊆ *N*; *W*_*i*_ represents the payment for the commission of the crime to each member *i* of the association *A* ⊆ *N* according to their criminal capacity *Pcg*_*i*_; *C* represents the payment for bribes; and the term $Pr(\sum_{\left(i,j\right)\in E_{A}}d_{ij})$ is the probability of information leakage, which depends on the total distrust in the association given by $\sum_{\left(i,j\right)\in E_{A}}d_{ij}$, where *E*_*A*_ ≔ {(*i*, *j*) ∈ *E*; *i*, *j* ∈ *A*}.

The use of this function in the formulation of an integer linear programming model to search for an association requires the definition of the following decision variables:

$$\begin{array}{c} X_{ij}=\{\begin{array}{cc} 1 & \text{if}\;\left(i,j\right)\;\;\in E_{A}\\ 0 & \text{otherwise}\; \end{array} \end{array}$$ (13)

$$\begin{array}{c} Y_{i}=\{\begin{array}{cc} 1 & \text{if}\;i\;\;\in A\\ 0 & \text{otherwise}\; \end{array} \end{array}$$ (14)

where Eq (13) indicates whether the link between individuals *i* and *j* belongs to the association and Eq (14) indicates whether individual *i* is part of the association. The planner’s linear utility function is:

$$\begin{array}{c} U=I\frac{\sum_{i\in N}Pcg_{i}Y_{i}}{Pcg_{max}}-w\sum_{i\in N}Pcg_{i}Y_{i}-I\gamma \frac{\sum_{(i,j)\in E}d_{ij}X_{ij}}{d_{max}} \end{array}$$ (15)

where *Pcg*_*max*_ is the maximum criminal propensity that the planner can consider in carrying out a crime; *d*_*max*_ is the greatest mistrust between the planner *s* and receiver *d*; *w* is the fee that the crime planner is willing to pay per unit of criminal capacity *Pcg*; and *γ* is the percentage of proceeds *I* intended for paying bribes.

We assume that under the worst association scenario, that is, for *Pcg*_*max*_ and *d*_*max*_, the profit obtained by the crime planner should be at least equal to the proportion of income corresponding to their criminal capacity *Pcg*_*s*_. This is denoted by the following equation:

$$\begin{array}{c} I-w\left(Pcg_{max}-Pcg_{s}\right)-I\gamma =I\frac{Pcg_{s}}{Pcg_{max}} \end{array}$$ (16)

Solving for *w* and replacing it in Eq (15), the crime planner’s utility function to search for an association is expressed as follows:

$$\begin{array}{c} U=I\gamma (\frac{\sum_{i\in N}Pcg_{i}Y_{i}}{Pcg_{max}-Pcg_{s}}-\frac{\sum_{(i,j)\in E}d_{ij}X_{ij}}{d_{max}}) \end{array}$$ (17)

## 3 Model to detect criminal groups starting with one suspect

We propose a novel model for detecting criminal groups starting with a single node, i.e., with a single suspect. Section 3.1 presents the proposed model. Section 3.2 illustrates how the model works using a simple artificial data set.

### 3.1 Model formulation based on Steiner trees

Our model is based on the following elements:

- The *SPWST* as introduced in Section 2.1
- The planner’s utility function as presented in Section 2.2, where in our case the planner will represent our only suspect.
- Each individual’s propensity for belonging to a criminal group as an attribute of the respective node
- The social distance between two individuals

As mentioned above, the proposed model considers the search for individuals belonging to a criminal group using the *SPWSTP*. The *SPWSTP* is represented by Eqs (4) to (8) [20] and a utility function characterized by Eq (17), transforming the problem into a net-worth maximization problem [21]. This tree should consider the root node *r*, restricting it as a node into which no edge enter. Given that the crime planner expects the value of income *I*, the incorporation of members into their grouping is restricted by the proportion of *I* that he or she is willing to distribute among the members. This proportion is defined by *φ* ∈ [0, 1] and allows generating different scenarios varying the number of individuals belonging to the group, thus providing a strong analysis tool.

Considering decision variables (1), (2), and (3) and the aforementioned restrictions, the proposed model called the Steiner Tree Prosecutor (*StPro*) is as follows:

$$\begin{array}{c} Max\;\;U=\frac{\sum_{i\in N}Pcg_{i}Y_{i}}{Pcg_{max}-Pcg_{r}}-\frac{\sum_{(i,j)\in E}d_{ij}X_{ij}}{d_{max}} \end{array}$$ (18)

s.t.

$$\begin{array}{c} \sum_{i\in N}X_{ij}=Y_{j}\;\forall j\in N\backslash \{s\} \end{array}$$ (19)

$$\begin{array}{c} \sum_{i\in N}f_{ij}-\sum_{i\in N}f_{ji}=Y_{j}\;\forall j\in N\backslash \{r\} \end{array}$$ (20)

$$\begin{array}{c} f_{ij}\leq \left(|N|-1\right)X_{ij}\;\forall i\in N,j\in N\backslash \{r\} \end{array}$$ (21)

$$\begin{array}{c} \sum_{i\in N}Pcg_{i}Y_{i}\leq \varphi Pcg_{max} \end{array}$$ (22)

$$\begin{array}{c} Y_{r}=1 \end{array}$$ (23)

$$\begin{array}{c} X_{ij}\in \{0,1\}\;\forall (i,j)\in E \end{array}$$ (24)

$$\begin{array}{c} Y_{i}\in \{0,1\}\;\forall i\in N \end{array}$$ (25)

$$\begin{array}{c} f_{ij}\geq 0\;\forall (i,j)\in E \end{array}$$ (26)

Constraints (19) to (21) generate the structure of the *SPWSTP*. Constraint (22) represents the fact that in making this choice, the planner is willing to select a proportion *φ* of the maximum criminal propensity that the planner can consider to carry out a crime. As is shown in our application (see Section 4), parameter *φ* provides a strong tool for analyzing different crime scenarios. Constraint (23) establish the only suspect. Constraints (24) and (25) are the binary and (26) non-negativity constraints.

To obtain *Pcg*_*max*_ and *d*_*max*_, we solve the maximum spanning tree problem (*MSTP*) [27]. This problem consists of finding the tree that covers all the nodes *N* ∈ *G* at maximum cost, rooted in *r*. In this way, an estimate of the structure of the worst criminal group that could form *r* is obtained by considering all the individuals in the network. This guarantees that each numerator of the objective function is never greater than its respective denominator. The values of *Pcg*_*max*_ and *d*_*max*_ are calculated as the sum of the solutions for nodes *Pcg*_*i*_ and edges *d*_*ij*_, respectively. Considering decision variables (13), (14), and (3), the formulation of the *MSTP* is as follows:

$$\begin{array}{c} Max\;\;\sum_{(i,j)\in E}d_{ij}X_{ij} \end{array}$$ (27)

s.t.

$$\begin{array}{c} \sum_{i\in N}\sum_{j\in N\backslash \{r\}}X_{ij}=|N|-1 \end{array}$$ (28)

$$\begin{array}{c} \sum_{j\in N}f_{rj}=|N|-1 \end{array}$$ (29)

$$\begin{array}{c} \sum_{i\in N}f_{ij}-\sum_{i\in N}f_{ji}=1\;\forall j\in N\backslash \{s\} \end{array}$$ (30)

$$\begin{array}{c} f_{ij}\leq \left(|N|-1\right)X_{ij}\;\forall i\in N,j\in N\backslash \{r\} \end{array}$$ (31)

$$\begin{array}{c} \sum_{i\in N}X_{ij}=Y_{j}\;\forall j\in N\backslash \{r\} \end{array}$$ (32)

$$\begin{array}{c} Y_{r}=1 \end{array}$$ (33)

$$\begin{array}{c} X_{ij}\in \{0,1\}\;\forall (i,j)\in E \end{array}$$ (34)

$$\begin{array}{c} Y_{i}\in \{0,1\}\;\forall i\in N \end{array}$$ (35)

$$\begin{array}{c} f_{ij}\geq 0\;\forall (i,j)\in E \end{array}$$ (36)

The main output of the model is a criminal network structure, which identifies the group of individuals most likely to be part of a criminal organization based on their propensity for crime and their social distance to the crime planner. Specifically, the output consists of:

- A Steiner tree representing the subset of individuals connected to the crime planner, forming the potential criminal group.
- The optimal distribution of the crime planner’s resources (*I*) among the selected individuals, controlled by the parameter *φ*, which determines the number of participants in the criminal network.
- The overall utility (*U*) of the criminal network, as calculated by the objective function Eq (18), which maximizes the net-worth of the crime planner while minimizing social distance between members and maximizing their propensity to commit a crime.

This output allows the crime planner to optimize their criminal group by balancing the potential gains from crime with the associated social risks, thus enabling scenario analysis for different values of *φ*.

### 3.2 Example application

**Fig 1:**
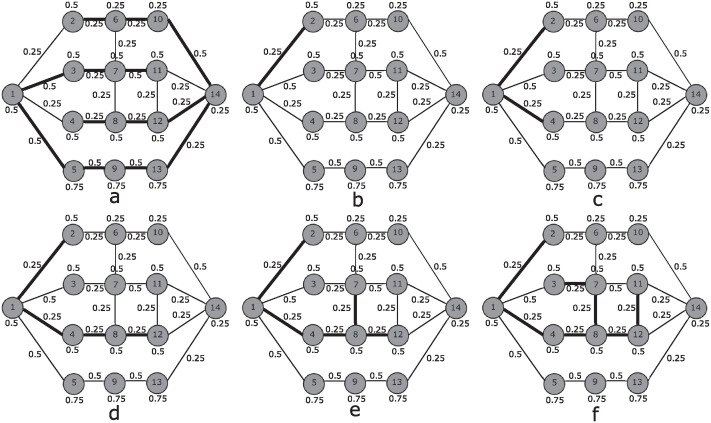
Example network.

To explain the operation of *StPro*, we consider the network shown in Fig 1a.

To apply *StPro* and to find the groupings starting with node 1, it is necessary to determine the parameters *Pcg*_*max*_ and *d*_*max*_ solving the *MSTP*. The value obtained for *Pcg*_*max*_ is 7 and for *d*_*max*_ is 4.75. The resulting spanning tree is indicated by bold edges in Fig 1a where the number next to an edge indicates its social distance (mistrust) and the number next to a node its propensity to belong to a criminal group.

Eq (37) shows the objective function of *StPro* for this example.

$$\begin{array}{c} Max\;\;U=\frac{\sum_{i\in N}Pcg_{i}Y_{i}}{7-0.5}-\frac{\sum_{(i,j)\in E}d_{ij}X_{ij}}{4.75} \end{array}$$ (37)

Varying parameter *φ*, the proposed model *StPro* provides different results using this objective function and *Pcg*_*max*_ = 7 in constraint (22). These are indicated by bold edges starting in Fig 1b.

Applying *StPro* with *φ* = 0.1 leads to the solution shown in Fig 1b. Node 2 and node 4 have the same distance to node 1 and the same propensity. Our model chooses arbitrarily node 2. For *φ* = 0.2 *StPro* adds node 4 as can be seen in Fig 1c. *φ* = 0.3 leads to the solution in Fig 1d where nodes 8 and 12 enter the respective solution. Fig 1e results for *φ* = 0.4. *StPro* extends the branch from node 8 to node 7. Finally, for *φ* = 0.5 the solution shown in Fig 1f is obtained. The branch previously extended to node 7 now connects to node 3.

## 4 StPro: Application and test of effectiveness

Next, we demonstrate the effectiveness of *StPro*. To do so, we apply this model to a network of criminals provided by the crime analysis unit of the Public Prosecutor’s Office of Chile.

### 4.1 Description of the data set

**Fig 2:**
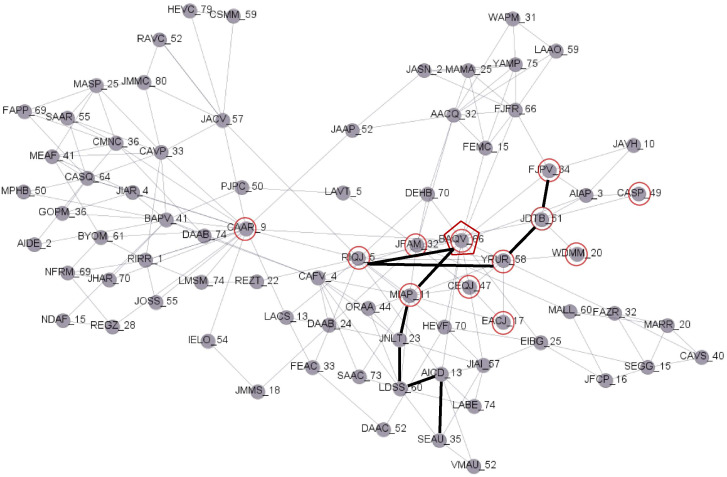
The network of 77 suspects.

The Public Prosecutor’s Office of Chile is an organization that directs criminal investigations by enforcing the law. It does this based on the evidence and historical data of the individuals accused of a crime, also called the suspects. Historical data describe the criminal behavior of a suspect and their links with other criminals, which is essential for obtaining networks for the investigation of criminal groups. The criminal network is obtained from a historical database of 1,666 offenses committed in the period 2002-2017 and 77 suspects. Within this network, there is a criminal group of burglars of uninhabited places investigated and identified in 2018. Fig 2 shows the criminal network, where the 12 members of the criminal group are identified by circles.

To analyze the properties of the criminal network, we computed several network metrics that provide insight into the overall structure and behavior of the network. These metrics include the network’s connectivity, the number of nodes and edges, the number of connected components, the maximum path length, the average and maximum degree, and the average clustering coefficient. Table 1 summarizes the most relevant metrics of the network, providing a comprehensive view of its topology.

**Table 1:** Network metrics.

Metric	Value
Connectivity	Connected
Number of nodes	77
Number of edges	374
Number of connected components	1
Maximum average path length	3.14
Average degree	9.71
Average clustering coefficient	0.4380

Network metrics are widely used to analyze complex networks, offering insights into the structure and behavior of criminal networks [8]. The clustering coefficient measures the tendency of suspects to form tightly connected groups, while the degree distribution highlights the most central individuals. Connectivity indicates whether all suspects are part of a single connected structure, and the number of nodes, edges, and components helps assess the size and fragmentation of the network. The maximum path length (diameter) reflects the longest distance between any two suspects, and the average/maximum degree identifies key individuals based on their number of connections. These metrics are essential for understanding the network’s structure and interaction patterns.

The analysis of the suspects’ network shows that all 77 individuals are connected, suggesting they may be part of a larger structure. With 374 edges, the network is fairly dense, and on average, each suspect is linked to nearly 10 others. However, some suspects stand out, as indicated by the maximum degree of 34 connections, suggesting they could be key figures within the group. The average path length between suspects is 3.14, with the longest path being 5, meaning that information or influence can spread quickly within the network. The clustering coefficient of 0.4380 suggests that some suspects tend to form well-connected subgroups, which could indicate the presence of a criminal organization within the broader network.

**Fig 3:**
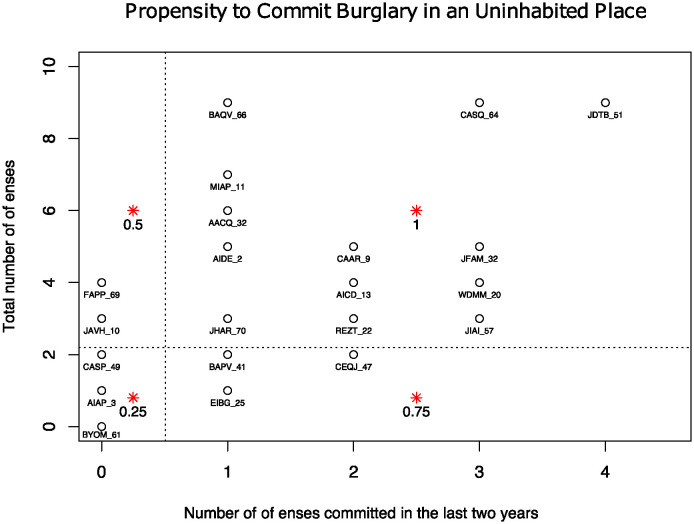
Classification of the 77 suspects, to estimate their propensity to commit burglary in an uninhabited place.

Fig 3 shows the criminal propensity values assigned to each member of the network. The suspects are classified according to the total number of offenses committed and the number of offenses committed during 2016 and 2017. The number of offenses during the years 2016 and 2017 expresses the most recent criminal energy for each individual whereas the total number of offenses indicates their overall criminal activities.

### 4.2 Test of the effectiveness of StPro

The effectiveness test for *StPro* consists of measuring its ability to identify members of a criminal group of burglars of uninhabited places within a criminal network for *φ* = 0.1, 0.2, 0.3, 0.4, and 0.5. Values of *φ* no greater than 0.5 are used. These low values make the number of individuals included in the solution very high and the solution less useful. The performance measures considered are precision and recall which are defined as follows:

$$\begin{array}{c} Precision=\frac{\text{Number}\;\text{of}\;\text{members}\;\text{of}\;\text{the}\;\text{criminal}\;\text{group}\;\text{in}\;\text{the}\;\text{solution}}{\text{Number}\;\text{of}\;\text{suspects}\;\text{in}\;\text{the}\;\text{solution}} \end{array}$$ (38)

$$\begin{array}{c} Recall=\frac{\text{Number}\;\text{of}\;\text{members}\;\text{of}\;\text{the}\;\text{criminal}\;\text{group}\;\text{in}\;\text{the}\;\text{solution}}{\text{Total}\;\text{Number}\;\text{of}\;\text{members}\;\text{of}\;\text{the}\;\text{criminal}\;\text{group}} \end{array}$$ (39)

The F-measure is the harmonic mean of Precision and Recall [28] and is defined as shown in Eq 40.

$$\begin{array}{c} F-measure=\frac{2\;Precision\;Recall}{Precision+Recall} \end{array}$$ (40)

For this test, *StPro* considers each one of the twelve members of the criminal group as the only suspect to start the respective police investigation. In total, there are 60 runs of the model (5 values for *φ* and 12 different criminal groups). To the best of our knowledge, there are no other models to detect criminal groups starting with just one single suspect.

As a consequence, we compare the performance of *StPro* with a modified shortest-path algorithm called *SPA* [15] and an integer linear programming model called *LiRAM* [14].

The *SPA* algorithm is a link analysis technique that uses shortest-path algorithms to identify the strongest association paths between entities in a criminal network. For this, a network is established in which the relationship between individuals is represented by a social bond interpreted as the probability that two individuals are linked. Independence between the links is assumed, and the association between two individuals is defined as the route of greatest probability. The transformation of the links through the negative natural logarithm allows this route to be obtained through the shortest path algorithm. *LiRAM* seeks the route between two individuals that maximizes the utility function between one of the individuals who is assigned the role of the crime planner. For this, a network is established in which the relationship between individuals is represented by a social distance, and in the nodes, the propensity of an individual to belong to a criminal group. *LIRAM* and *SPA* need at least a pair of suspects. Since they consider more information (two suspects), it is to expect that both outperform *StPro*. However, we will show that this is not the case.

To apply *LiRAM* and *SPA*, pairs of individuals are selected under the context that two members of the criminal group not directly related have already been identified. Eighty-six pairs are identified that are applied for the five values of *φ*.

*StPro* and *LiRAM* were implemented using the AMPL language and solved using CPLEX 22.1.1.0 solver. *SPA* Was implemented in R. The system specifications included an 11th Gen Intel(R) Core(TM) i7-11800H 2.30 GHz processor with 16.00 GB of RAM.

In Fig 2, the thick line shows the solution found by *StPro* between individuals considering the root individual *BAQV*_66 for *φ* = 0.2. Within this solution, nine individuals are identified, not including *BAQV*_66, of which five are members of the criminal group. The precision, recall, and F-measure are as follows:

$$\begin{array}{c} Precision=\frac{5}{9}=0.555 \end{array}$$ (41)

$$\begin{array}{c} Recall=\frac{5}{12}=0.416 \end{array}$$ (42)

$$\begin{array}{c} F-measure=\frac{2*0.555*0.416}{0.555+0.416}=0.4755 \end{array}$$ (43)

**Fig 4:**
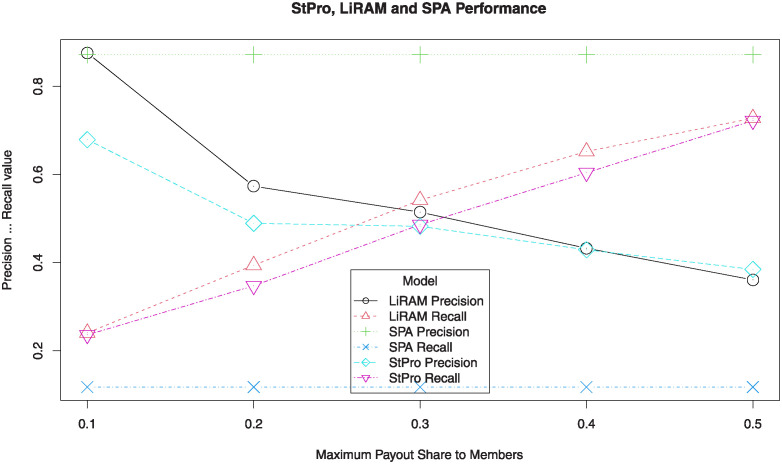
Precision and recall of StPro, LiRAM, and SPA.

Fig 4 shows the precision and recall values for *StPro*, *LiRAM*, and *SPA*. We can see that *StPro* provides an average precision and average recall similar to those of *LiRAM* for each value of *φ* and greater than the constant values of *SPA*.

**Fig 5:**
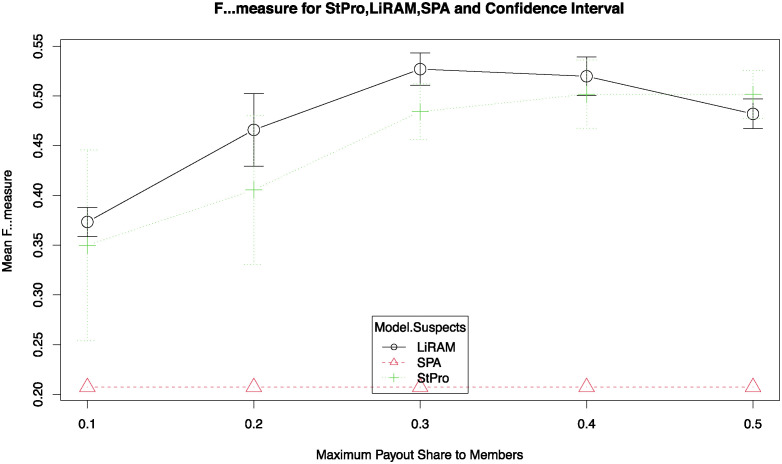
F-measure to StPro, LiRAM and SPA.

Fig 5 shows the average F-measure values for *StPro*, *LiRAM*, and *SPA* with confidence intervals for a confidence level of 95%. *StPro* shows a slightly lower performance than *LiRAM* for each value of *φ*. Fig 5 also shows that the confidence intervals intersect, so it is evident that there may not be a significant difference between the results of both models.

To determine if the difference between both models is statistically significant for each value of *φ*, a test of the hypothesis of the difference in means between the models is applied. To define the test to be used, the normality and homogeneity in the variance of the results of both models is studied for each value of *φ*. The normality was studied using the Shapiro–Wilk test. Table 2 shows the results of this test for each model and the value of *φ*.

**Table 2:** Results of statistical tests for different values of *φ*.

Test	Maximum Payout Share to Members P-value				
	*φ* = 0.1	*φ* = 0.2	*φ* = 0.3	*φ* = 0.4	*φ* = 0.5
**Shapiro-Wilk (LiRAM data)**	3.41e−9	2.16e−3	0.00278	0.0117	0.239
**Shapiro-Wilk (StPro data)**	0.0250	0.003742	0.00201	0.0401	5.6471e−4
**Levene**	6.102e−5	0.1438	0.05163	0.03224	0.03611
**Kruskal-Wallis**	0.8504	0.4327	0.07577	0.6481	0.2407

According to Table 2, the results of the Shapiro–Wilk test for a significance level of 0.05 allow rejection of the null hypothesis; that is, the results are not normally distributed, except for the results of *LiRAM* for a value *φ* = 0.5. Given the lack of normality in the data and given that the groups are not all of equal size, the Levene test is applied to study the homogeneity in the variance. The results are shown in Table 2. The null hypothesis is rejected for all groups at a significance level of 0.05, concluding that there is no homogeneity in the variance.

This lack of normality and homogeneity in the variance and the presence of extreme data prevent the application of the traditional parametric analysis of variance (ANOVA) test to evaluate the difference in means. Therefore, the non-parametric Kruskal–Wallis test is applied, which tests whether the analyzed data sets come from the same population [29]. Table 2 shows the results of the Kruskal–Wallis test for the different values of *φ*. For a significance level of 0.05, it is not possible to reject the null hypothesis, so we conclude that the results of both models are statistically similar. This result implies that starting a police investigation considering a single suspect and applying *StPro* allows us to obtain results as good as those obtained by *LiRAM* starting with two suspects.

## 5 StPro in apprehending criminal gangs in Chile

**Fig 6:**
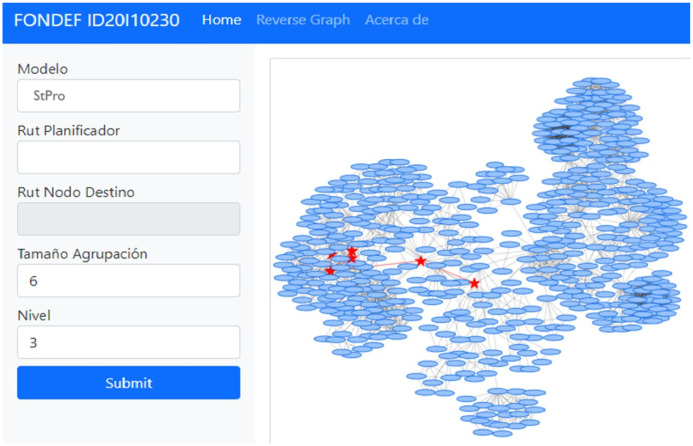
Current implementation of StPro.

StPro is currently being implemented as a prototype at the Criminal Analysis Unit of the National Prosecutor’s Office of Chile. The system’s current developmental stage is depicted in Fig 6, which visualizes the network of individuals to be analyzed (blue nodes) and the resulting tree generated by *StPro* (red star nodes). The search parameters include the Planner (Rut Planificador), representing the root of the tree; Cluster Size (Tamaño Agrupación), determining the number of individuals considered in the tree; and Level (Nivel), which limits the network’s diameter.

On Friday, May 12, 2023, news broke that *StPro* played a crucial role in apprehending 10 individuals, aged between 15 and 22, who belonged to a criminal gang, called “Influencers” involved in two types of property crimes commonly known in Chile as “encerrona” and “portonazo.” These crimes exhibited similar characteristics and occurred in different locations within the Metropolitan Region of Santiago, Chile [30]. “Encerrona” involves forcibly stopping a car and stealing it using one or more vehicles, while “portonazo” entails stealing a car as it stops at its destination, typically the victim’s residence. Both crimes are highly violent and have a significant social impact.

The identification process of the criminal gang commenced with preliminary information gathered by the Chilean Investigative Police (PDI), which identified four individuals as suspects in the case: *Suspect*_1_, *Suspect*_2_, *Suspect*_3_, and *Suspect*_4_.

**Fig 7:**
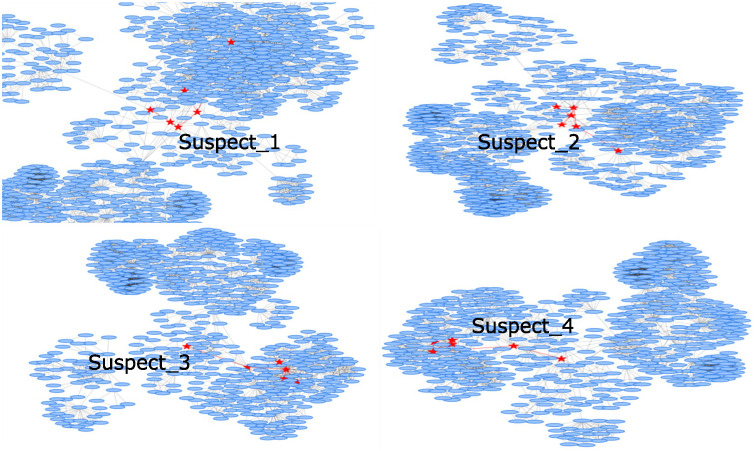
Trees generated by StPro for each of the four suspects.

*StPro* was independently applied to each suspect using a cluster size of 6 and level 3. The four resulting trees are displayed in Fig 7.

Through the analysis of the *StPro*-generated trees, eight new suspects were identified. By utilizing photographic records, all 12 suspects were positively identified by their victims, leading to their subsequent appearance at a detention control hearing. The process of formal charges and trial will be carried out by other units within the National Prosecutor’s Office of Chile.

## 6 Conclusions and future work

This article introduces *StPro*, a novel approach for detecting criminal groups starting with just one suspect, contributing to the disruption of criminal networks. It is based on the node-weighted *STP* and integrates methods from Social Network Analysis. To the best of our knowledge, existing models for identifying criminal groups require at least two suspects, which limits their effectiveness in real-world investigations where many cases begin with only one suspect. By addressing this limitation, *StPro* offers a powerful tool for disrupting criminal groups by enabling investigators to map connections and identify key individuals, even with minimal initial data.

A criminal utility function was used in the adaptation of the node-weighted *STP* to enhance the detection of criminal groups. Its application to a real-world crime analysis demonstrates *StPro*’s potential in criminal investigations, yielding excellent results. It performs comparably to models requiring two suspects, showing that this approach can deliver impactful outcomes with less information. The identification of key members within a criminal gang further underscores *StPro*’s strong capability to support investigations aimed at disrupting criminal organizations.

*StPro* opens new pathways for applied research in crime investigation and disruption. For future work, the model will be applied sequentially, with updates to model parameters and re-analysis each time a new suspect is confirmed. Additionally, exploring its application without confirmed suspects and relying on the propensity of potential suspects could further enhance its utility for proactively disrupting criminal networks.
